# P-1679. Neutrophil-related Cell Population Data as an Immediate Biomarker for the Assessment of Therapeutic Response in Methicillin-Resistant Staphylococcus aureus (MRSA) Bacteremia

**DOI:** 10.1093/ofid/ofaf695.1853

**Published:** 2026-01-11

**Authors:** Daisuke Ono, Yusuke Nishida, Kazuyuki Mimura, Kunihisa Tsukada, Hideaki Oka, Yasufumi Suzuki, Masumi Ogawa, Hiromi Kataoka, Kyosuke Takeshita

**Affiliations:** Saitama Medical Center, Ota, Gumma, Japan; Saitama Medical Center, Ota, Gumma, Japan; Saitama Medical Center, Ota, Gumma, Japan; Saitama Medical Center, Ota, Gumma, Japan; Saitama Medical Center, Ota, Gumma, Japan; Saitama Medical Center, Ota, Gumma, Japan; Saitama Medical Center, Ota, Gumma, Japan; Kawasaki University of Medical Welfare, Kurashiki, Okayama, Japan; Saitama Medical Center, Ota, Gumma, Japan

## Abstract

**Background:**

MRSA bacteremia requires early anti-MRSA therapy, as beta-lactams are ineffective; delays for pathogen identification and susceptibility results can worsen outcomes. Rapid biomarkers are thus essential. Cell population data (CPD), captured via flow cytometry by automated hematology analyzers, appears earlier than conventional laboratory changes but is not reported to clinicians. CPD is readily available at no extra cost and shows promise for early exacerbation detection, though its utility remains underexplored.

**Figure 1:**
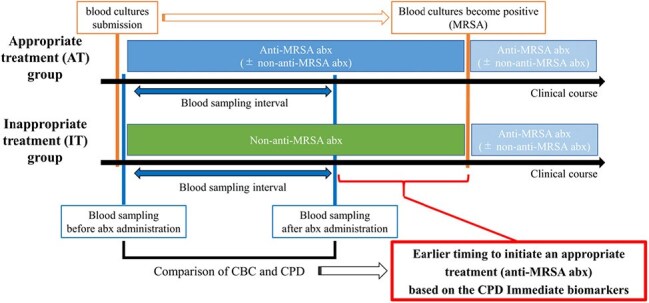
Study Flowchart for Classification of MRSA Bacteremia Patients Based on Initial Antimicrobial Therapy to Evaluate the Utility of Cell Population Data (CPD) Abbreviations: Abx, antibiotic; CBC, complete blood count

**Table 1:**
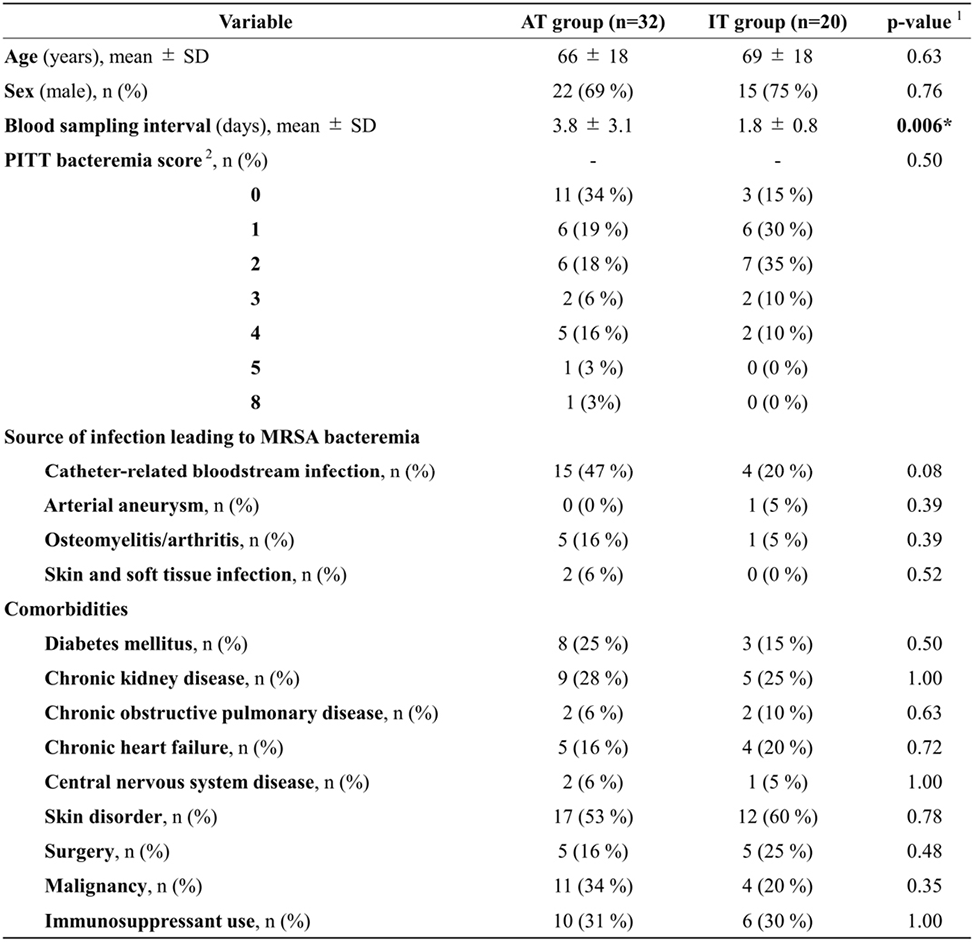
Comparison of Patient Characteristics Between the Appropriate Therapy Group (AT Group; Anti-MRSA Agents Initiated) and the Inappropriate Therapy Group (IT Group; Non-Anti-MRSA Antibiotics) 1. Statistical analyses were performed using Stata/BE 18 (StataCorp., College Station, TX, USA). Continuous variables were analyzed using the t-test, and binary or categorical variables using Fisher’s exact test. A two-sided p-value of <0.05 was considered statistically significant. Variables showing statistical significance are indicated with an asterisk. 2. Battle SE, et al. Prediction of mortality in Staphylococcus aureus bloodstream infection using quick Pitt bacteremia score. J Infect. 2022 Feb;84(2):131–5.

**Methods:**

We retrospectively analyzed MRSA bacteremia cases at Saitama Medical Center, a tertiary hospital in Japan (April 2018–March 2021). Patients were grouped into appropriate therapy (AT; anti-MRSA agents) or inappropriate therapy (IT; non-anti-MRSA agents) (Fig. 1). Hematologic parameters, including CPD from XN-9000 analyzers (Sysmex Corp., Japan), were collected before and after antibiotic initiation; post-/pre-treatment ratios were calculated to minimize interpatient variability. Univariate analyses assessed patient characteristics and laboratory value ratios between groups. Logistic regression adjusting for significant covariates evaluated therapy appropriateness. A multivariable model yielded a composite score, and its diagnostic performance was evaluated.

**Table 2:**
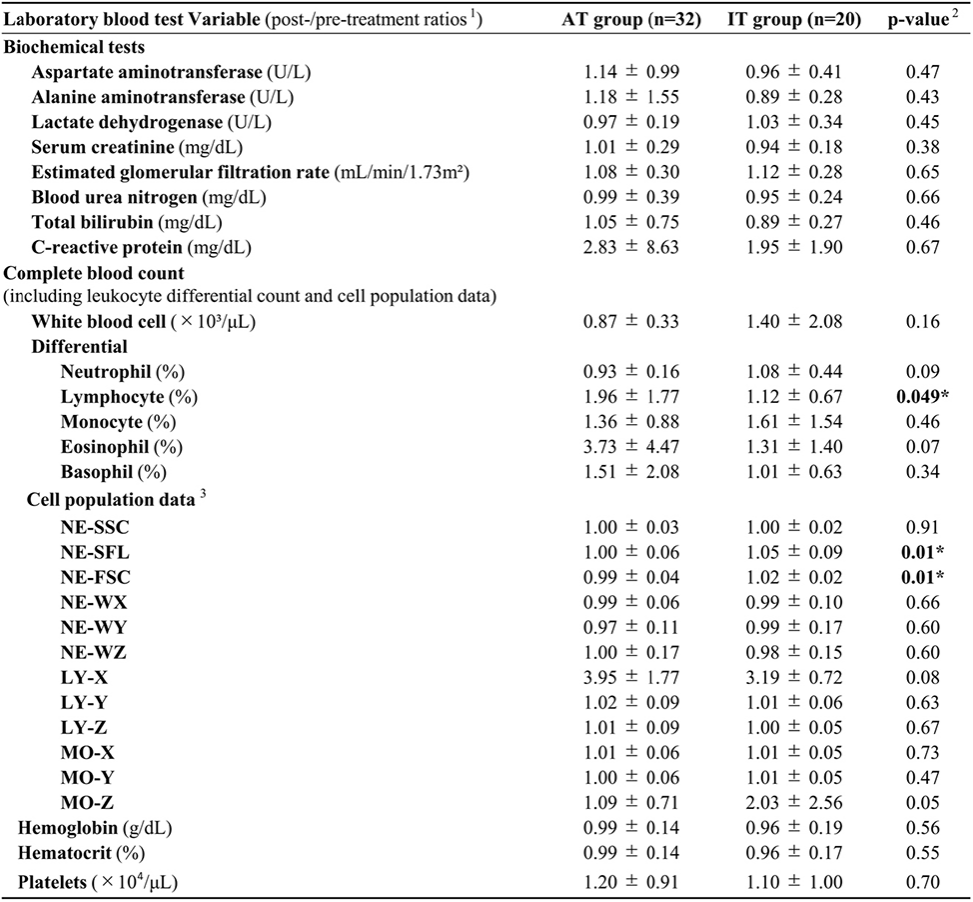
Comparison of Patient Characteristics Between the Appropriate Therapy Group (AT Group; Anti-MRSA Agents Initiated) and the Inappropriate Therapy Group (IT Group; Non-Anti-MRSA Antibiotics) 1. Hematologic parameters, including cell population data (CPD) from XN-9000 analyzers (Sysmex Corp., Kobe, Japan), were collected before and after antibiotic initiation, and post-/pre-treatment ratios were calculated to reduce interpatient variability. 2. Statistical analyses were performed using Stata/BE 18 (StataCorp., College Station, TX, USA). Continuous variables were analyzed using the t-test. A two-sided p-value of <0.05 was considered statistically significant. Variables showing statistical significance are indicated with an asterisk. 3. The prefixes NE-, LY-, and MO- indicate that the corresponding CPD are derived from neutrophils, lymphocytes, and monocytes, respectively.

**Figure 2:**
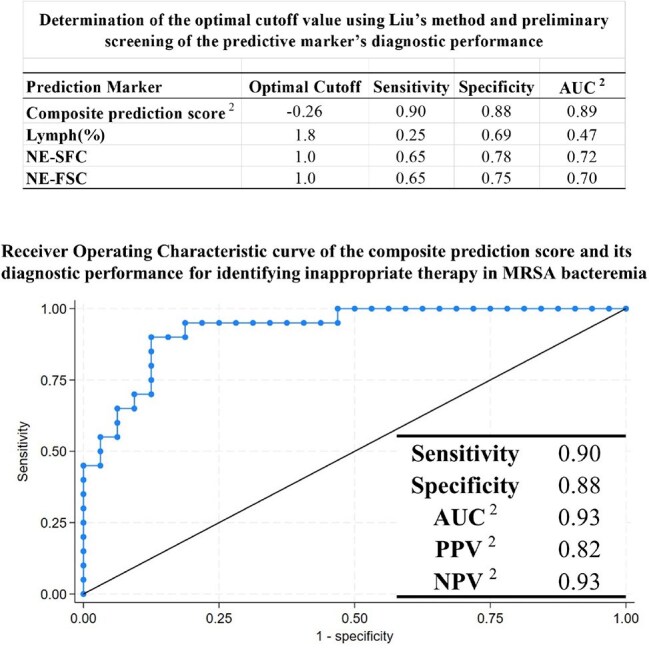
Diagnostic Performance of a Composite Prediction Score for Identifying Appropriate Versus Inappropriate Therapy in MRSA Bacteremia 1 The composite prediction score was derived from the following multivariable logistic regression model: The composite prediction score = β_0_ + β_1_ × lymphocyte (%) + β_2_ × NE-SFL + β_3_ × NE-FSC + β_4_ × interval_1_ + β_5_ × interval_2_ β_0_ = intercept value β_1_ = coefficient for lymphocyte (%) β_2_ = coefficient for NE-SFL β_3_ = coefficient for NE-FSC, β_4_ = coefficient for restricted cubic spline term (blood sampling interval_1_) β_5_ = coefficient for restricted cubic spline term (blood sampling interval_2_) To screen for the optimal cutoff of the composite prediction score, Liu’s method was applied. The final diagnostic performance of the composite prediction score was then derived from the receiver operating characteristic curve based on the Liu’s method-derived cutoff. 2. Abbreviations: AUC, Area Under the Receiver Operating Characteristic Curve; PPV, Positive Predictive Value; NPV, Negative Predictive Value.

**Results:**

Fifty-two patients (AT 32, IT 20) were analyzed. Univariate analysis identified four significant factors: blood sampling interval and changes in lymphocyte (%), NE-SFL, and NE-FSC (both CPD parameters) (Table 1, 2). No strong multicollinearity was observed among these three markers (r = −0.11 to +0.35). A composite model from multivariable logistic regression adjusted for the interval and including lymphocyte (%), NE-SFL, and NE-FSC showed excellent discrimination (AUC 0.93, 95 % CI: 0.86–1.0), with sensitivity 0.90, specificity 0.88, PPV 0.82, and NPV 0.93 (Fig. 2).

**Conclusion:**

Neutrophil-related CPD parameters quickly reflected treatment appropriateness in MRSA bacteremia. A composite score derived from lymphocyte (%), NE-SFL (nucleic acid content), and NE-FSC (cell size) may enable early recognition of ineffective therapy. As CPD is routinely collected without added cost or procedures, it offers a practical biomarker to prompt timely anti-MRSA adjustments.

**Disclosures:**

All Authors: No reported disclosures

